# Public Awareness and Use of Price Transparency: Report From a National Survey

**DOI:** 10.2196/64439

**Published:** 2024-12-12

**Authors:** Yuvraj Pathak, David Muhlestein

**Affiliations:** 1 West Health Institute La Jolla, CA United States; 2 Simple Healthcare Sanford, FL United States

**Keywords:** price transparency, consumer choice, survey, questionnaire, finance, cost, economics, price, pricing, consumer, transparent, Medicare, Medicaid, insurance

## Introduction

It is well known that health care spending in the United States is much higher than similar countries but that this does not result in better health outcomes [1]. Research has shown that this higher spending is driven by high prices rather than greater utilization of care [2]. Until recently, publicly available information on health care prices has been scarce [3,4]. This began to change when the hospital price transparency (HPT) went into effect on January 1, 2021 [5].

HPT requires hospitals to post prices or negotiated rates of common, “shoppable,” services, as defined by the Centers for Medicare and Medicaid (CMS), in a machine-readable format. Examples of these shoppable services include common blood tests and radiology services such as X-rays and mammograms. In July 2022, the CMS also mandated that payers post the prices for a more expansive set of services, under the Transparency in Coverage (TIC) regulation [6]. Thanks to HPT and TIC, health care prices are no longer hidden from the public.

CMS’s stated goal [5,7] for these transparency initiatives is to help inform consumers about the cost of medical services prior to receiving them, as this information can help people make informed decisions about where they receive services. Additionally, transparency about prices could lead to greater competition and potentially lower prices and health care spending.

These transparency initiatives can be impactful if people are aware that they can access information on health care prices for decision-making, especially for shoppable services with comparable quality across providers. This impact is only actualized if hospitals comply with CMS requirements to provide complete and accurate data on prices in a timely manner.

This paper aims to examine whether people are aware that they can look up prices of health care services prior to receiving care, and whether this newly available information is being used.

## Methods

### Overview

The data for this study come from a nationally representative survey conducted by Gallup in partnership with West Health Institute from November 2023 through January 2024 that included 5149 randomly selected adults aged 18 years and older from across all 50 states and the District of Columbia from the Gallup Panel [8].

### Ethical Considerations

The survey received ethical approval from Gallup’s internal institutional review board for all human subject and external reporting considerations (2023-10-04). Participation in the survey was voluntary. All the responses were deidentified and no personal health information or other sensitive personal information was shared with the authors. Web respondents were offered an incentive of $5 while mail respondents were offered an incentive of $2.

### Survey

The survey response rate was 38%. Further details on the sampling strategy are in Multimedia Appendix 1. The survey consisted of questions on people’s experiences and perceptions of health care in America. For this study, we focus on four questions: respondents’ (1) awareness of, (2) use of, and (3) and (4) opinions about compliance with price transparency laws. Percentages were estimated as the count of responses divided by the number of respondents, multiplied by 100. Probability sampling weights provided by Gallup were used to weight the estimated percentages and to calculate the population estimates.

## Results

### Low Awareness of Price Transparency

Almost three-quarters of respondents (73%) were not aware that they could look up prices for common health care services provided by hospitals. Men were less likely (77%) than women (70%) to be aware of HPT. Lack of awareness was similar across all ages (Table 1).

**Table 1:** Awareness of price transparency.

Characteristics			Responses, %				Population size, N	
			Yes		No			
**Are you aware that hospitals are required to post prices on their website?**								
	Full sample (n=4946)		27		73		248,092,303	
	**Gender**							
		Male (n=2513)		23		77		124,047,066
		Female (n=2371)		30		70		118,640,004
	**Race**							
		White (n=3091)		28		72		194,419,832
		Non-White^a^ (n=1827)		23		77		51,826,750
	**Age (years)**							
		18-29 (n=345)		27		73		43,876,733
		30-39 (n=619)		27		73		44,685,260
		40-49 (n=576)		25		75		41,625,267
		50-64 (n=1551)		25		75		60,433,949
		65 years and older (n=1855)		29		71		57,471,094
**Have you ever looked up the price of a healthcare service before going to a hospital** **?**								
	Full sample (n=4954)		19		81		248,212,278	
	**Gender**							
		Male (n=2523)		21		79		123,971,168
		Female (n=2369)		16		84		118,835,877
	**Race**							
		White (n=3096)		23		77		194,756,253
		Non-White (n=1830)		18		82		51,610,304
	**Age (years)**							
		18-29 (n=346)		29		71		43,614,198
		30-39 (n=620)		23		77		44,980,477
		40-49 (n=575)		20		80		41,553,530
		50-64 (n=1554)		16		84		60,512,956
		65 and older (n=1859)		11		89		57,551,117

^a^The category “non-White” is used because there was 1 race/ethnicity variable instead of 2 separate variables for race and ethnicity. The non-White group included a mix of races and ethnicities.

### Limited Use of Price Transparency Data

Less than 20% of respondents said they had looked up the price of a medical service prior to their visit. Younger respondents (age 18-29 years; 29%) were more likely than older respondents (aged ≥65 years; 11%) to have ever looked up a price prior to going to a hospital.

### Opinions About Enforcing Compliance

Overall, 78% of respondents preferred stricter penalties for hospitals that have not yet released their prices, and only 26% were in favor of CMS allowing hospitals more time to comply with the HPT requirements (Table 2).

**Table 2:** Opinions on enforcement of price transparency rules.

Characteristics				Responses, %							Population size, N
				Disagree or strongly disagree		Neutral		Agree or strongly agree			
**The federal government should impose and enforce stronger penalties on the hospitals that have not made their negotiated prices public.**											
	Full sample (n=5004)			5		17		78		249,963,323	
	**Gender**										
		Male (n=2534)	7		19		75		124,394,088		
		Female (n=2408)	4		15		81		120,164,001		
	**Race**										
		White (n=3122)	6		16		78		195,617,984		
		Non-White^a^ (n=1854)	5		18		78		52,499,618		
	**Age (years)**										
		18-29 (n=348)	3		18		79		44,314,231		
		30-39 (n=617)	5		13		81		44,620,889		
		40-49 (n=575)	5		15		79		41,571,949		
		50-64 (n=1571)	6		17		77		61,030,674		
		65 and older (n=1893)	6		18		75		58,425,579		
**The federal government should allow more time for hospitals to comply before strictly enforcing the law.**											
	Full sample (n=4958)			51		23		26		248,828,600	
	**Gender**										
		Male (n=2510)	46		26		28		123,838,600		
		Female (n=2386)	56		20		23		119,584,767		
	**Race**										
		White (n=3102)	54		23		23		195,228,742		
		Non-White (n=1828)	40		24		36		51,754,137		
	**Age (years)**										
		18-29 (n=347)	45		24		31		44,180,940		
		30-39 (n=618)	56		20		23		44,916,392		
		40-49 (n=576)	57		23		20		41,625,267		
		50-64 (n=1557)	55		20		24		60,693,140		
		65 and older (n=1860)	45		27		28		57,412,860		

^a^The category “non-White” is used because there was 1 race/ethnicity variable instead of 2 separate variables for race and ethnicity. The non-White group included a mix of races and ethnicities.

## Discussion

Increased price transparency has the potential to increase competition and decrease health care spending in the United States [3,4,9]. It can also help consumers of health care make informed decisions [5]. We found limited awareness and use of HPT data among the public, but there is a strong desire for transparency in health care prices. It should be noted that these findings are based on self-reported data and might differ from actual awareness and use of HPT. The CMS should improve compliance among hospitals by aggressively enforcing the existing penalties [10]. Public awareness efforts could also lead to increased use by consumers.

## Appendix

Multimedia Appendix 1 Sampling strategy and survey questions.

## Abbreviations

CMS: Centers for Medicare and Medicaid
HPT: hospital price transparency
TIC: Transparency in Coverage
