# Supplementary material for: Public Awareness and Use of Price Transparency: Report From a National Survey
Source: Interact J Med Res. 2024 Dec 12;13:e64439. doi: 10.2196/64439 (PMC11671781; doi:10.2196/64439)
Supplement: Multimedia Appendix 1 [file ijmr_v13i1e64439_app1.docx]

**Sampling Strategy**

This survey was fielded via web and mail to 5,149 adults aged 18 and older in all 50 states and the District of Columbia from November 13, 2023, to January 8, 2024, using the Gallup Panel. The mail portion of the survey was focused on older Americans aged 50 and older. The Gallup Panel is a probability-based panel of U.S. adults that is recruited using digital-dial (RDD) phone interviews and address-based sampling methods (ABS). At the conclusion of the RDD and ABS surveys, respondents are asked to agree to being re-contacted for future Gallup surveys. Approximately 80% agree and are eligible for recruitment into the panel.

80,000 Panel members have provided necessary information to be contacted for web, mail, and telephone surveys. Another 20,000 members can be reached for mail and telephone surveys. receive an average of three surveys per month.

The average response rate on a Gallup Panel survey is approximately 40-45%. For this survey, the response rate was 38%. Web respondents were offered an incentive of $5 while mail respondents were offered an incentive of $2.

A stratified sample design was employed to collect the sample. 19 mutually exclusive strata were created, and samples were randomly selected from within each of the strata. Older adults age 50+ were oversampled to increase the stability of results for this population segment.

The sampling error is ±1.7% (±1.0 %) at 95% confidence interval for response percentages around 50 % (90%). Respondent knowledge and consent was obtained for all respondents and the final data was weighted to correct for non-response to match national level demographics as per Current Population Survey.

**Questions on Price Transparency**

Beginning in 2021, the federal government requires hospitals to post information about the prices that they have negotiated with insurers for common medical services such as a consultation or lab test.

WH401 Are you aware of this law that requires hospitals to post prices of medical services on their website?

- Yes (1)
- No (2)
- Don’t know/No opinion (3)

WH402 Have you looked up pricing for a healthcare service before going to a hospital or doctor’s office?

- Yes (1)
- No (2)
- Don’t know/No opinion (3)

WH403 Some hospitals have still not made their pries publicly available. How much do you agree or disagree with the following statements?

**[PROGRAMMER NOTE: Rotate A-B]**

1. The federal government should impose and enforce stronger penalties on the hospitals that have not made their negotiated prices public.
2. The federal government should allow more time for hospitals to comply before strictly enforcing the law.

- Strongly agree (1)
- Somewhat agree (2)
- Neither agree nor disagree (3)
- Somewhat disagree (4)
- Strongly disagree (5)
